# The role of conformity in mask-wearing during COVID-19

**DOI:** 10.1371/journal.pone.0261321

**Published:** 2021-12-17

**Authors:** Anna Woodcock, P. Wesley Schultz

**Affiliations:** California State University San Marcos, San Marcos, California, United States of America; VIT University, INDIA

## Abstract

By September 2020, COVID-19 had claimed the lives of almost 1 million people worldwide, including more than 400,000 in the U.S. and Europe [1] To slow the spread of the virus, health officials advised social distancing, regular handwashing, and wearing a face covering [2]. We hypothesized that public adherence to the health guidance would be influenced by prevailing social norms, and the prevalence of these behaviors among others. We focused on mask-wearing behavior during fall 2020, and coded livestream public webcam footage of 1,200 individuals across seven cities. Results showed that only 50% of participants were correctly wearing a mask in public, and that this percentage varied as a function of the mask-wearing behavior of close and distant others in the immediate physical vicinity. How social normative information might be used to increase mask-wearing behavior is discussed. “Cloth face coverings are one of the most powerful weapons we have to slow and stop the spread of the virus—particularly when used universally within a community setting” CDC Director Dr. Robert Redfield in July 2020.

## Introduction

During its first six months, the global COVID-19 pandemic killed more than a million people. Reliable data about the contagiousness of the virus and exactly how it was transmitted was initially scarce and conflicting, but by June 2020 both the Centers for Disease Control (CDC) and the World Health Organization (WHO) were sending clear and consistent messages that behavioral measures such as staying at least 6-feet (~2 meters) from others who don’t live with you, regularly washing your hands, and wearing a facial mask covering your nose and mouth were effective ways to curb the transmission of the virus. By September 2020 more than eight-in-ten U.S. adults (85%) reported wearing a mask all or most of the time when they were inside stores or businesses, and 74% of U.S. adults reported always wearing a face mask when they were outside of their homes [3,4]. Despite an initial slowing during widespread lockdown and stay-at-home orders, COVID-19 infections continued to grow, and by October 2020 had been transmitted to more than 7.4 million Americans, killing 210,000. As the world awaited safe and effective vaccines, individual behaviors were the frontline defense against the spread of COVID-19.

Despite evidence from scientific and global public health data about its effectiveness, wearing a mask or cloth facial covering became a source of contention in the U.S. and elsewhere. Statistical models of per capita COVID-19 mortality rates during the onset of the pandemic showed that deaths were highest in regions that were slow to adopt mask-wearing mandates. This was further illustrated by data regarding the low levels of coronavirus mortality seen in Asian countries that adopted widespread public mask usage early in the outbreak, either via mandate or social norms [5].

In the United States and abroad, legal mandates were the strongest predictor of mask-wearing, but there was also systematic variation by level of education, gender, age group, race/ethnicity, and political affiliation. Survey data showed that:

- In the U.S., college graduates were more likely to report having worn a mask all or most of the time (76%), compared with those without a college degree (60%).
- Women (87%) were more likely than men (83%) to report wearing a mask.
- Adults over 65 reported wearing masks at higher rates than younger age-groups.
- Whites were less likely to report wearing masks than other racial and ethnic groups.
- Democrats were more likely than Republicans to report wearing masks (76% vs. 53%), and conservative Republicans were among the least likely to say they have worn a mask all or most of the time (49%) compared with 60% of moderate Republicans [3,6].

Psychologists have stressed that psychological factors are essential to understanding and responding to COVID-19. European data reveal that psychological factors such as perceptions of risk, the observed behavior of others, concern for self-protection and protecting others, and perceptions of the judgment of others when wearing a mask were also found to be significant predictors of mask wearing [7,8]. Self-reported concerns about the behavior and judgement of others suggests that social norms may influence decisions about when, where and with whom to wear a mask in public. A large body of evidence from social psychological research attests to the power of normative information about close others and individuals in the immediate vicinity to shape individual attitudes and behavior [9–12]. While the majority of Americans claimed to regularly wear a mask in public spaces, they report substantially lower rates of mask-wearing by others. For example, only 44% of Americans said that all or most of the people in their area have been wearing masks in stores or other businesses [6].

Prior research has established the causal influence of normative beliefs on behavior [13]. From classic research on conformity, to more contemporary research on normative beliefs and social marketing, behavioral research has shown that perceptions about the common and accepted behaviors of others can produce conformity and pressure to align one’s behavior with that of a group [10]. Research on normative social influence has been conducted across a range of behavioral domains, including environmental protection, gambling, paying taxes, and health behaviors such as alcohol and tobacco use. While the influence of social norms has been well-established, the current COVID-19 situation provided an opportunity to study a novel behavior (mask-wearing) in a context where the behavior had both a direct personal benefit (self-protection) and social benefit (slowing the spread of the virus).

Observation of public mask-wearing and the rate of mask-wearing in the immediate social context can advance our understanding of normative social influence, and directly inform efforts to promote wide-spread behavioral adoption. Two overarching questions guided this research: how often do adults correctly wear a mask in public spaces, and to what extent are people influenced by the mask-wearing behavior of proximal others? Specifically, we predicted that observed mask-wearing would be significantly lower than self-reported rates, and positively associated with the behavior of both proximal others and distant others in the immediate vicinity.

## Methods

Restricted guidelines covering human subjects research during COVID-19 require novel approaches to collecting behavioral data. This study used structured field observations of mask-wearing behavior via livestream public webcams, with IRB approval from California State University San Marcos (CSUSM). Given the nature of collecting observational data, informed consent was waived.

### Location

**Fig 1:**
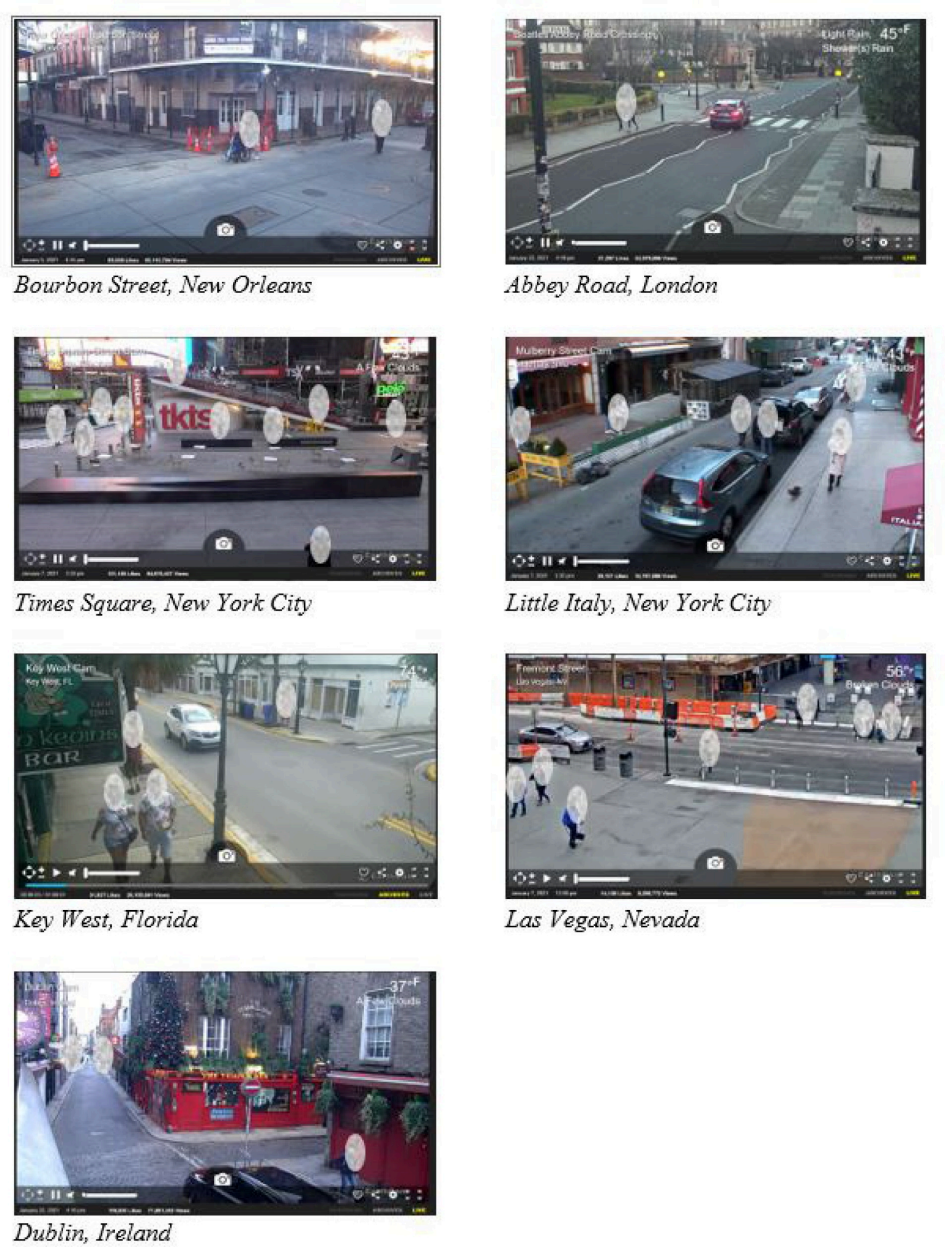
Livestream webcam locations.

Nine livestream public webcams across the U.S., the U.K., and Ireland were selected as locations for data collection. These locations represented all available livestream public webcams positioned such that the mask-wearing behavior of participants entering the frame was clearly visible (www.earthcam.com). Data were successfully collected from seven locations: 1) Bourbon Street, New Orleans; 2) Abbey Road, London; 3) Times Square, 4) and Little Italy, New York City; 5) Duval Street, Key West; 6) Essex Gate, Dublin; and 7) Freemont Street, Las Vegas (see Fig 1). Webcams in Miami and New Jersey were also selected but the cameras were not operational during the data collection period. All locations were urban streetscapes in high pedestrian traffic areas; at the time of observation, the U.S. locations had city-wide mandates to wear a mask indoors and outdoors in public spaces [14–18] while wearing masks was only mandated indoors for London and Dublin [19,20].

### Measures

#### Demographics

For each focal participant, we coded estimated age range, sex, and race/ethnicity from the webcam footage and screenshots.

#### Mask-wearing

The mask-wearing behavior of the focal participant and each person in the screenshot frame were coded as: mask worn correctly (completely covering the nose and mouth), mask worn incorrectly or no mask worn, mask present but not worn (because of eating, smoking etc.), or unknown if the person was not facing the camera.

#### Foot traffic and groupings

The density of the foot traffic during each observation was coded as: sparse (65%), moderate (25%), or heavy (10%). Focal participants were coded as being alone, part of a pair, or part of a group, and their actions were coded as walking, sitting, or movement such as cycling or jogging.

#### Location

Each webcam automatically recorded current weather conditions (e.g., mostly sunny, light rain, cloudy, etc.), temperature, and local time and date.

### Procedure

Twenty-four trained research assistants each recorded 50 independent observations between September 9^th^– 15^th^, 2020. Groups of research assistants were assigned to specific webcams and made their observations at non-overlapping time periods. All training and coding were conducted online to comply with State and county stay-at-home orders. The research assistants were trained as a group via zoom with sample images across all nine webcams, and then in smaller groups with live feed from their assigned webcams.

Observations were made of a focal participant, any individuals accompanying the focal participant, and individuals in the immediate vicinity. Several features of the physical location, time and date, and weather conditions were also captured. During an observation session, coders logged in to the webcam and checked for clear weather conditions and the presence of foot traffic. Focal participants were randomly selected by the roll of a 6-sided die or a 1–6 random number generator. The *n*th adolescent or adult to move into the frame was designated as the focal participant for that observation. Coders watched the focal participant for approximately 5-seconds and then downloaded a screen shot from the webcam system. The live observation and screenshot details were coded and entered onto a coding sheet. Once one observation was completed and coded, the coder drew another random number and coded the next focal participant.

#### Inter-rater reliability

To maximize coding reliability all coders completed a 1-hour group training session via zoom that included independently coding 12 sample webcam screenshots and coming to a consensus via group discussion. Observers were assigned to webcam locations in groups of four and they coded live footage and screenshots of their location and reaching a consensus before the formal data collection began. Formal tests of inter-rater reliability (IRR) were assessed with data from a sample of 14 coders who coded 19 randomly selected screenshots from webcams other than their own. One-way random average-measures Intraclass Correlation Coefficients (ICCs) assessed the consistency in their ratings on the key variables. The resulting ICCs were in the good—excellent range; estimated age group = .984, estimated sex = 1.0, mask-wearing = 1.0, density of foot traffic = .952, and masks worn in groups = .993 indicating that coders had a high degree of agreement. The consensus about the number of masks worn in the broader vicinity was lower at .687, and the observation of race/ethnicity did not reach a statistically acceptable level, ICC = .346, which was to be expected [21].

### Participants

A target of 50 observations was set for each of the 24 coders, across nine locations (*N* = 1,200). Using conservative estimates of effect size, *a priori* power analyses indicated that a total sample size of 950 was required to detect a small effect of *d* = 0.2, with 85% power for the planned independent means t-tests, and 1,093 was required to detect a small effect of *w* = 0.1, with 85% power for planned chi-square tests [22]. We successfully collected data from 1,150 individuals (focal participants) across the nine public access live feed webcams (www.earthcam.com). Data from two defective webcams were removed, leaving an analytic sample of 1,140 observations from seven webcams. Visual assessment of focal participants concluded that 55.4% were male, 63% were White/Caucasian, 9.4% Latinx, 16% Black/African American, 4.3% Asian, and the remainder could not be determined. Age group was coded as teens and young adults (< 30 yrs.) 43.4%, adults 30–50 years of age 43.4%, and adults over the age of 50 accounted for 13.2%. Eighty-nine percent of focal participants were walking, 8.2% were cycling, skateboarding, or running, and 2.8% sitting or in a wheelchair. Forty-eight percent of focal participants were alone, 31% were with one other person (referred to as a pair from here), and the remaining 21% were in a group with at least two other people. Eighty percent of observations were made on weekdays, split across the afternoon (48%), the evening (33%) and 19% in the late night or morning local time. The observations were taken on fine clear days with good visibility; mean temperature of 77 degrees Fahrenheit (55–98 range).

## Results

### Overall mask-wearing

Despite contemporaneous national survey data showing that 74% of American adults claimed to wear a mask in public, we observed that only 51% of focal participants across the seven locations were correctly wearing a mask covering their nose and mouth. Forty-six percent were not wearing a mask, and 3% had a mask around their neck or in their hand or were engaged in an activity that temporarily precluded them from wearing a mask (e.g., eating or drinking). We noted that none of the webcams captured visible signage encouraging mask-wearing or social distancing. A chi-square goodness-of-fit test revealed that the observed incidents of mask-wearing was statistically significantly lower than the self-reported rates of 74%, *χ*^*2*^(1) = 315.63, *p* < .001. Our analyses of mask-wearing are restricted to focal participants in the no mask or mask worn incorrectly (coded 0), and mask worn correctly (coded 1) categories.

**Fig 2:**
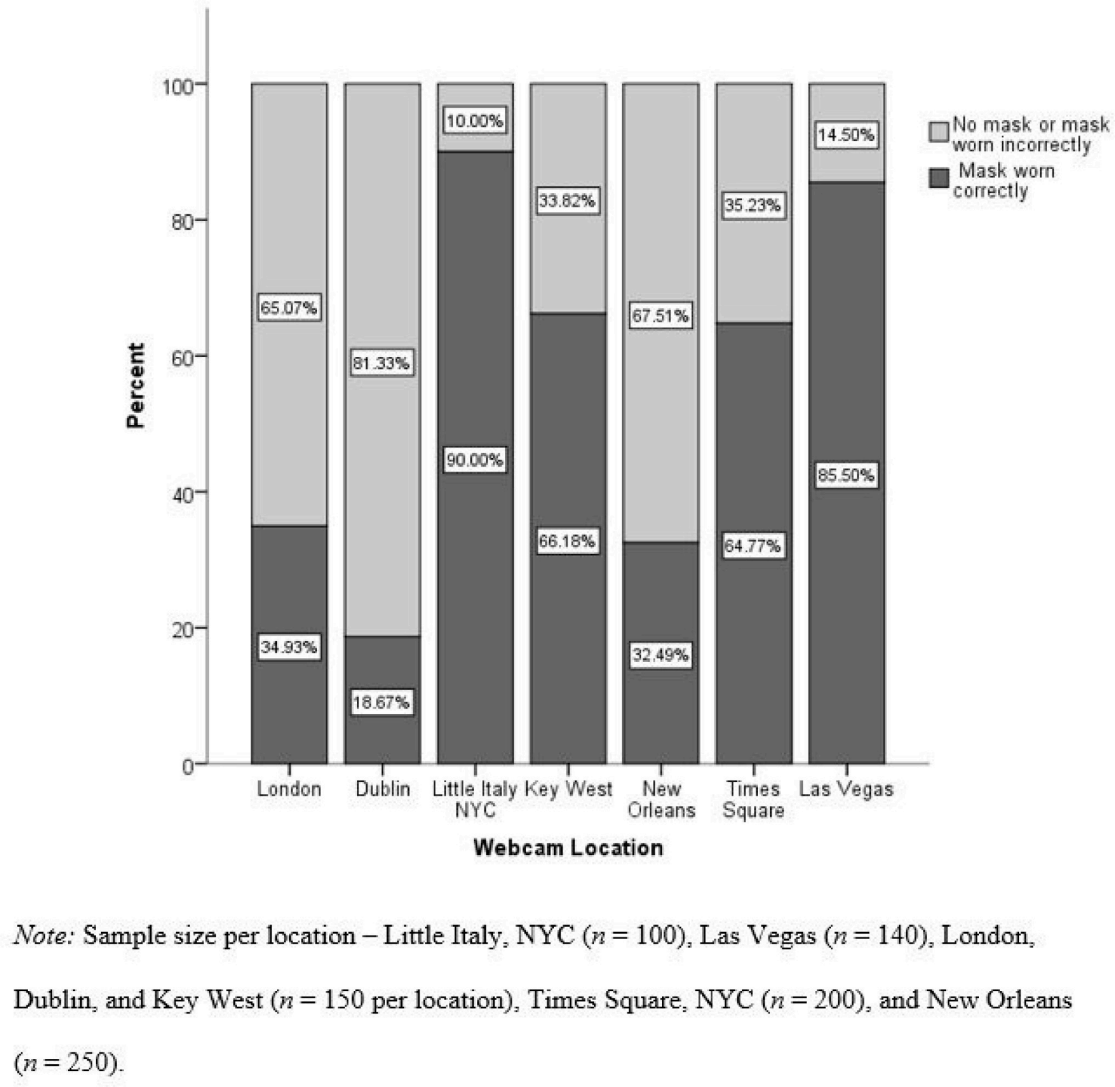
Mask-wearing by location.

Mask-wearing differed significantly across location, ranging from 90% of focal participants wearing a mask correctly in New York’s Little Italy, to fewer than 20% in Dublin (See Fig 2). The intraclass correlation coefficient (ICC) across the seven sites was .49 indicating variability both within and between locations. Mask-wearing was more common in the U.S. locations (59%) where mask-wearing was mandated for outdoor public spaces compared with the European locations where mask mandates applied only to indoor public spaces (26%), *χ*^*2*^(12) = 29.95, *p* < .001. However, the observed rate of 59% of the U.S. participants still fell short of the expectation of 74% obtained from survey data, *χ*^*2*^(1) = 30.70, *p* < .001. In line with national survey data, mask-wearing differed by estimated sex, with 58% of females compared with 48% of males correctly wearing a mask, *χ*^*2*^(1) = 12.38, *p* < .001. Mask-wearing also differed as a function of the density of the crowd (and ability to social distance), with 70.3% of participants wearing a mask in a crowded situation, compared to moderately or sparsely crowded situations (45% and 53% respectively), *χ*^*2*^(2) = 18.92, *p* < .001. Mask-wearing did not vary by age group in our sample, *χ*^*2*^(2) = 3.97, *p* = .137.

### Mask-wearing in groups of close others

Our first test of the influence of the behavior of others on mask-wearing compared participants observed in public alone and with proximal others. Counter to our expectations, mask-wearing did not differ significantly between people in a public space alone, as part of a pair, or part of a group *χ*^*2*^(2) = 1.125, *p* = .570. However, the mask wearing behavior of focal participants in a pair was influenced by their partner’s mask-wearing. Eighty-three percent of the 342 focal participants in a pair had the same mask-wearing status as their partner: 41% both wearing masks and 42% both with no masks.

To explore the effect of mask-wearing behavior in groups, we calculated the percentage of people (other than the focal participant) in each group who were wearing a mask correctly. Two hundred and twenty-six focal participants were with groups of two or more other people. If the social norms communicated by groups of close others influenced mask-wearing behavior, we would expect focal participant mask-wearers to be in groups containing significantly higher percentages of mask-wearers than focal participants who were not wearing a mask. We found that focal participants who were wearing a mask correctly were in groups with an average of 84% mask-wearers; focal participants who were not wearing a mask were in groups who averaged just 31% mask-wearers, *t*(169) = 10.76, *p* < .001, *95% CI* of the difference in means [4.26, 6.23], *d* = 1.38.

### Mask-wearing in the proximity of unknown others

We took a similar approach to examine the impact of the mask-wearing behavior of the people in the larger social context. We calculated the percentage of people (other than the focal participant) in the immediate vicinity who were wearing a mask. Given the impact of close others on mask-wearing reported above, we chose to examine the influence of the behavior of others in the immediate vicinity on the mask-wearing behavior of our 549 focal participants who were in public by themselves. The number of other people in the immediate social context ranged from 0 to 59, *median* = 7.00. We limited our analyses to focal participants with at least one other person in the vicinity, and to the people in the immediate vicinity for whom mask-wearing could be determined. This reduced the range from 1 to 41 people, *median* = 2.00. While not as large as the effect of close others, we found that the mask-wearing behavior of proximal unknown others had a statistically significant effect on mask-wearing. Focal participants who were wearing a mask correctly were in social contexts with an average rate of 33% mask-wearers; focal participants who were not wearing a mask were in social contexts with an average rate of 19% mask-wearers, *t*(475.64) = 4.91, *p* < .001, *95% CI* of the difference in means [0.08, 0.19], *d* = 0.46.

## Discussion

In the months following the start of the worldwide COVID-19 pandemic, the primary tool for slowing the viral spread and protecting public health involved changes in individual behavior. Among the behaviors recommended by health professionals, one of the most important was wearing a mask in public. Our analyses of the mask-wearing behavior of individuals across seven U.S. and European cities showed that significantly fewer individuals correctly wore a mask covering their mouth and nose while in public spaces than was reported by national surveys.

Our reported results examined the role of descriptive norms on mask-wearing behavior. Across the seven public sites included in our study, the intraclass correlation coefficient (ICC) was .49. The ICC is calculated as the ratio of variability between groups, divided by the sum of the variability both within and between groups. An ICC of zero would indicate no variability between groups, and a high degree of variability within groups. In contrast, an ICC of 1.0 would indicate variability between groups, but no variability within groups. In our current situation, the ICC of .49 indicates that there was more variability between groups than would have occurred at random.

We interpret this as evidence for conformity. However, an alternative explanation is that individuals within a group (in our case, cityscape) are affected by the physical and social aspects of their surroundings, including political inclinations of the region, local restrictions, and national mandates. Thus, the observed ICC across the selected sites may be due to shared features of the site, rather than to social norms. However, importantly, our reported results also showed clustering of behavior within each site. Focal individuals in groups tended to match the mask-wearing tendencies of the rest of the group. Mask wearers were in groups with an average mask-wearing rate of 84%, while those without a mask were in groups with an average mask-wearing rate of 34%. This second finding lends more support for our conformity interpretation.

This second finding showing a greater tendency for the mask-wearing behavior of focal individuals to match that of others in close proximity can also be explained as homophily—that is, the tendency for individuals to self-select into groups based on similar preferences. However, our third finding provides some additional support for our conformity interpretation in that distant others in the same setting showed behavioral similarity. We found that focal participants who were wearing a mask correctly were in social contexts that averaged 33% mask-wearers; focal participants who were not wearing a mask were in social contexts that averaged just 19% mask rates.

Our results suggest that an individual’s decision to engage in behaviors that can help slow the spread of a virus—such as wearing a face mask in public—has an important social component. Despite the clear personal and social benefits associated with wearing a face mask in public, a substantial number of individuals in our observations of public spaces chose *not* to wear a mask. These findings have important implications for efforts to promote health protective behaviors. The primary tool used by health official during the pandemic focused on protecting oneself and others. While such messages were influential—as indicated by the 51% of observed individuals wearing masks in our observation sample—these health-based messages ignore an important social consideration.

Based on our findings, public health communications that highlight the large number of individuals who are wearing masks can go a long way toward creating a support context for the target behaviors. Indeed, messages that highlight the large number of individuals who deviate from the norm have been shown in other studies to reduce conformity [12]. Instead, images and messaging highlighting widespread support for targeted public health measures can serve to amplify the protective personal and social health benefits. Similarly, messages that highlight the trending norm of increased behavioral adoption have also been found to promote higher rates of conformity [23,24].

In conclusion, behavioral data collected from public livestream webcams demonstrates significantly lower rates of mask-wearing than indicated by national survey data. This underscores the need to promote mask-wearing, and strongly indicates that the behavior of close others and strangers in the social context may have a potent influence on the decision to wear a mask.
